# Memory Phenotype CD4 T Cells Undergoing Rapid, Nonburst-Like, Cytokine-Driven Proliferation Can Be Distinguished from Antigen-Experienced Memory Cells

**DOI:** 10.1371/journal.pbio.1001171

**Published:** 2011-10-11

**Authors:** Souheil-Antoine Younes, George Punkosdy, Stephane Caucheteux, Tao Chen, Zvi Grossman, William E. Paul

**Affiliations:** Laboratory of Immunology, National Institute of Allergy and Infectious Diseases, National Institutes of Health, Bethesda, Maryland, United States of America; National Jewish Medical and Research Center/Howard Hughes Medical Institute, United States of America

## Abstract

Memory phenotype (CD44^bright^, CD25^negative^) CD4 spleen and lymph node T cells (MP cells) proliferate rapidly in normal or germ-free donors, with BrdU uptake rates of 6% to 10% per day and Ki-67 positivity of 18% to 35%. The rapid proliferation of MP cells stands in contrast to the much slower proliferation of lymphocytic choriomeningitis virus (LCMV)-specific memory cells that divide at rates ranging from <1% to 2% per day over the period from 15 to 60 days after LCMV infection. Anti-MHC class II antibodies fail to inhibit the in situ proliferation of MP cells, implying a non–T-cell receptor (TCR)-driven proliferation. Such proliferation is partially inhibited by anti–IL-7Rα antibody. The sequence diversity of TCRβ CDR3 gene segments is comparable among the proliferating and quiescent MP cells from conventional and germ-free mice, implying that the majority of proliferating MP cells have not recently derived from a small cohort of cells that expand through multiple continuous rounds of cell division. We propose that MP cells constitute a diverse cell population, containing a subpopulation of slowly dividing authentic antigen-primed memory cells and a majority population of rapidly proliferating cells that did not arise from naïve cells through conventional antigen-driven clonal expansion.

## Abstract 2

Contrary to the current paradigm that nearly all memory T cells proliferate in response to antigenic stimulation, this paper shows that an important population of CD4 T lymphocytes achieves memory/effector status independent of antigenic stimulation.

## Author Summary

The class of immune cells called CD4 T lymphocytes consists of two major cell types: naïve cells that have not yet participated in an immune response and memory cells, which are cells that have responded to antigen, expanded in number, and acquired new characteristics. These two cell types can be distinguished from one another because they display different cell surface marker proteins. In this paper, we argue that many—probably most—of the cells researchers generally characterize as memory cells on the basis of their surface markers are not authentic memory cells. True memory cells—the ones produced, for example, when we immunize a child against a disease—divide very slowly, whereas the bulk of the cells we generally characterize as memory cells divide very rapidly. Mice that have never been exposed to antigens have as many of these “memory-like” cells as normal mice have, implying that these cells arise by a process that does not require foreign antigen. Analysis of the sequence of the antigen recognition receptors on these “memory-like” cells indicates that their replication does not derive from a few cells or clones undergoing multiple rounds of proliferation, thus their division cannot be explained by conventional, antigen-driven clonal expansion. We conclude that this large population of “memory-like” cells has arisen by a mechanism independent of a response to foreign antigen, and that these cells may have a crucial biological function.

## Introduction

Peripheral non-Treg CD4^+^ T cells are often divided into two major subpopulations that can be designated naïve-phenotype (NP) and memory-phenotype (MP) cells, respectively [1]. In the mouse, MP cells are characterized by the expression of high levels of CD44 and low levels of CD45RB; they lack Foxp3 and high levels of CD25. MP cells may be either CD62L dull or bright [2]. It is generally assumed that MP cells constitute the aggregate of all antigen-specific memory cells; that is, of all cells that have expanded in response to antigenic stimulation. However, there are some reasons to question the concept that all MP cells are indeed foreign antigen-experienced cells. MP cells proliferate rapidly; estimates of their proliferative rates in lymph nodes range from 4% to 10% per day [2],[3]. By contrast, T-cell receptor (TCR) transgenic [4],[5] or polyclonal [5],[6] CD4 T cells that had responded to immunization with cognate antigens or infection proliferate at <1% to 2.5% per day when examined after the initial expansion and contraction phases have been completed [7]. The proliferation of antigen-primed CD4 T cells is largely driven by cytokines rather than through TCR stimulation [8]–[14]. What drives the rapid, apparently spontaneous, proliferation of MP under normal conditions is unknown, although when transferred to lymphopenic recipients, their proliferation is burst-like (i.e., they divide multiple times in a relatively short period) and appears to be driven by TCR-mediated stimulation.

Understanding the proliferation of MP cells has also been of considerable interest among those studying lymphocyte dynamics in chronic infections, particularly with lentiviruses, where proliferative rates of human or macaque MP cells in HIV- or SIV-infected individuals are much greater than those of comparable cells from noninfected individuals [15],[16]. Indeed, such rapid proliferation has been associated with the state of excessive inflammation that, in turn, has been regarded as a principal driver of the immunodeficiency of AIDS patients [17]–[19]. It has been suggested, on the basis of BrdU labeling and of measurement of Ki-67 expression in SIV-infected macaque CD4 T cells, that much of the proliferation of these MP cells represents recent burst-like divisions, presumably in response to antigenic stimulation, of cells that were undergoing the familiar pattern of clonal expansion and transition from central or effector memory populations to tissue-seeking effector cells [17],[20]. Although this mode of proliferation appears to be the case for SIV-infected macaques and presumably HIV-infected humans, whether it explains the proliferation of MP cells in normal individuals has not been determined. Recognizing that MP CD4 T cells constitute a large and heterogeneous population, we repeated previous experiments establishing the differences in proliferative rates of MP cells from those of authentic antigen-experienced memory cells and also compared the behavior and frequency of MP CD4 T cells in conventional and germ-free (GF) mice. In order to understand whether the proliferation of MP cells in situ (not in transfer models) was driven by antigen and was burst-like or by cytokines and was stochastic, we treated mice with anti-MHC class II antibodies or with anticytokine antibodies. Further, we reasoned that if the expansion of MP cells was burst-like, it should have originated from a small number of precursors and thus proliferating MP cells should have a much more limited TCR sequence diversity than MP cells that were not dividing.

Our results indicate that in situ MP cell division is driven largely by cytokines and not by TCR-mediated stimulation, that the diversity of the CDR3 regions of TCR β chains of particular VβJβ sets is similar in dividing and nondividing cells, and that conventional and GF MP cells are not distinguishable in either frequency, division rate, or, in a preliminary analysis, in sequence diversity. These results imply that the bulk of MP CD4 T cells in young adult mice differ in key respects from authentic antigen-driven memory cells.

## Results

### CD4 MP T Cells Proliferate More Rapidly Than Antigen-Specific Memory Cells

**Figure 1:**
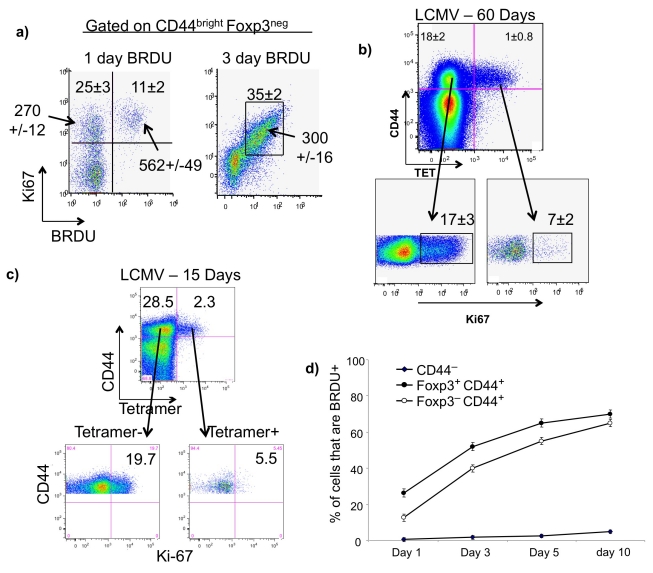
Rapid proliferation of MP cells. (A) Mice received a single IP injection of BrdU (1 mg) and were humanely killed 24 h later or received BrdU for 3 d in their drinking water (0.8 mg/ml) and were humanely killed at the end of the labeling period. Lymph node cells were collected and stained with anti-Foxp3, anti-CD4, anti-CD44, anti-Ki-67, and anti-BrdU. Numbers inside the quadrants are the mean frequencies of BrdU^+^/Ki-67^+^ and BrdU^−^/Ki-67^+^ cells (mean ± standard deviation [SD] for three replicate animals). Numbers with arrows are the MFI of Ki-67 staining (mean ± SD). (B) B6 mice were infected IP with 2 × 10^5^ plaque-forming unit (PFU) of LCMV Armstrong; 60 d later, spleen cells were stained with an I-A^b^-GP66-77 (DIYKGVYQFKSV) tetramer, anti-CD44, anti-CD4, and anti-Ki-67. In the upper panel, the mean frequency of CD44^bright^ cells among the tetramer+ and tetramer− cells from three replicates is shown (mean ± SD). The proportion of Ki-67^+^ cells among the CD44^bright^ tetramer− and tetramer+ cells is shown in the lower panel. (C) B6 mice infected with LCMV Armstrong 15 d earlier were analyzed for CD44 expression and tetramer binding. The lower panels represent the proportion of Ki-67^+^ cells among CD44^bright^ tetramer+ and tetramer− cells. (D) Normal B6 mice were placed on BrdU (0.8 mg/ml) in their drinking water for a period of 10 d. On days 1, 3, 5, and 10, mice were humanely killed and lymph nodes cells were collected and stained with anti-Foxp3, anti-CD4, anti-CD44, and anti-BrdU.

To readdress the question of the relative proliferative rates of MP cells and antigen-specific memory cells, we first evaluated the use of Ki-67 as a measure of recent proliferation. C57BL/6 mice received BrdU in a single intraperitoneal (IP) injection and were humanely killed 24 h later or BrdU was administered in their drinking water and mice were humanely killed 3 d later. Figure 1A shows that 11% of CD44^bright^ Foxp3^−^ CD4 lymph node T cells evaluated 24 h after a single injection of BrdU were stained by an anti-BrdU antibody, confirming the rapid proliferative rate of these cells. All of these BrdU^+^ cells were Ki-67^+^ and, in addition, 25% of the CD44^bright^ Foxp3^−^ CD4 cells were Ki-67^+^/BrdU^−^, as anticipated, since Ki-67 is known to be expressed for a period of time after cells have completed their cycle.

When we examined cells from the mice that had received BrdU for 3 d (Figure 1A), we found that 35% of the cells were BrdU^+^, reaffirming their rapid proliferative rate. The great majority of the Ki-67^+^ cells were BrdU^+^, indicating that most cells do not retain Ki-67 expression for more than 3 d after their last division. Indeed, Pitcher et al. [21] reached a similar conclusion regarding Ki-67 expression as a result of analyzing the proliferation of PBMCs from SIV-infected macaques by simultaneous staining for BrdU and Ki-67 [21]. Accordingly, we used either Ki-67 or BrdU in different experiments; particularly, we have relied on Ki-67 in later experiments in which we examined TCR Vβ sequence diversity among dividing and nondividing MP cells. In those experiments, we also took advantage of the finding (Figure 1A) that Ki-67 mean fluorescence intensity (MFI) was highest in cells that had taken up BrdU during the previous 24 h.

We then compared the frequency of Ki-67^+^ cells among splenic MP cells and antigen-specific (tetramer+) CD4 T cells obtained 60 d after acute lymphocytic choriomeningitis virus (LCMV) infection (Figure 1B). The frequency of tetramer+ CD4 T cells in LCMV-infected mice is greater among splenic CD4 T cells than lymph node CD4 T cells, so we limited our evaluation to tetramer+ cells from the spleen and compared them to splenic MP cells whose proliferative rates are somewhat less than those of lymph node MP cells. In this experiment, 17%±3% of the splenic MP cells were Ki-67^+^. Among tetramer+ CD4 T cells (5.2% of the CD44^bright^ CD4 T cells) obtained from mice infected 60 d earlier, only 7%±2% were Ki-67^+^. This finding implies that ∼2% of the tetramer+ cells divided each day, a frequency similar to the proliferative rates of antigen-specific memory CD4 T cells reported by others [5]–[7].

This difference in proliferative rates could be explained if MP cells have derived quite recently from NP cells and are still dividing relatively rapidly, while the tetramer^pos^ memory cells induced by intentional immunization were examined 60 d after infection, and in experiments reported by others at least 40 d after infection/immunization, when their proliferative rates may have slowed considerably.

If this were the case, we might anticipate that authentic memory cells would be dividing substantially more rapidly when studied relatively shortly after infection. We assessed the expression of Ki-67 in tetramer^pos^ and tetramer^neg^ CD44^bright^ CD25^−^ CD4 T cells 15 d after LCMV infection. At that time, 7.7% (±0.3%) of the CD44^bright^ cells were tetramer^pos^. Of these, 7.8%±1.9% were Ki-67^+^ compared to 18.9%±0.6% of the tetramer^neg^ MP cells (Figure 1C, results from one of three mice). This experiment indicates that one cannot account for the differences in the in situ proliferative rate of MP cells and of the antigen-driven memory cells on the basis of the more recent priming of the former than the latter. We did verify that tetramer^pos^ cells examined 6 d after infection were essentially all (97%) Ki-67^+^, indicating that these cells had undergone rapid proliferation as a result of antigenic stimulation. As we will show later, it is highly unlikely that most of the Ki-67^+^ MP cells represent a population in the midst of its antigen-driven expansion from naive or memory precursors.

The high proliferative rate of MP cells might be due to a distinct, small subpopulation dividing very rapidly while a large population divides slowly. We thought that explanation unlikely in view of the classic report by Tough and Sprent [2] that more than 60% of CD44^bright^ CD4 T cells became BrdU^+^ during a labeling period of 30 d, implying that over that period of time the great majority of CD44^bright^ CD4 T cells had divided at least once. We observed an even more rapid proliferation with ∼60% of MP (CD44^bright^ Foxp3^−^) CD4 T cells having taken up BrdU in a 10-d labeling period (Figure 1D), again arguing that the high proliferative rate of MP cells is not a property of a small subpopulation of these cells.

**Figure 2:**
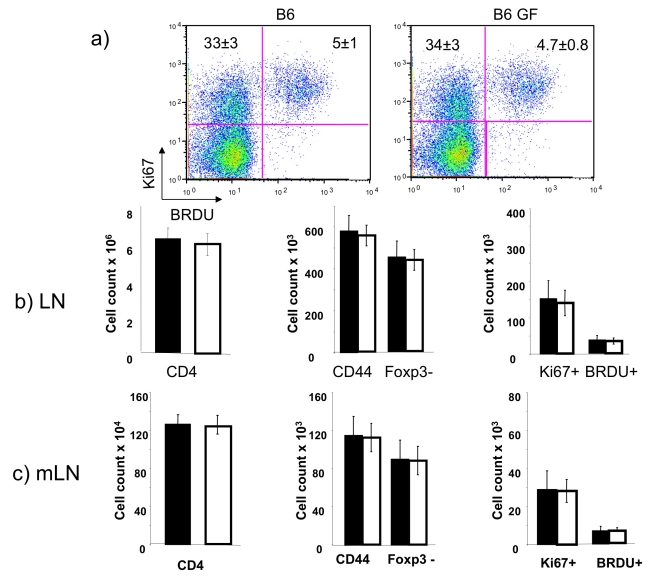
In situ proliferation of MP cells in GF mice. (A) Conventional B6 and B6 GF mice received an IP injection of 1 mg BrdU and were humanely killed 6 h later. Lymph nodes cells were collected and stained with anti-Foxp3, anti-CD4, anti-CD44, anti-BrdU, and anti-Ki-67. The numbers in the upper quadrants represent the mean percent of BrdU^+^ and Ki-67^+^ cells (mean ± SD for three replicate animals). (B and C) Numbers of CD4^+^, CD44^+^, CD44^+^ Foxp3^−^, Ki-67^+^, and BrdU^+^ cells in peripheral LN (B) and mesenteric LN (C) of conventional (black bars) and GF (white bars) mice.

The presence and proliferation of MP cells in GF mice needs to be considered in assessing the possible role of foreign antigens in stimulating the in situ proliferation of MP cells in normal animals. We reported that the proliferative rate of CD44^bright^ CD25^−^ cells in SW GF mice was ∼4% in 6 h and was no different from that of such cells from conventional SW mice [3]. This implies that the generation and proliferation of MP cells can be achieved in mice with very limited antigenic load. To examine this in greater detail and in the mouse strain that was being studied in our experiments, we injected BrdU into conventional and GF C57BL/6 mice and evaluated the frequency of BrdU^+^ cells 6 h later. BrdU^+^ cells constituted 4.7% of the GF CD44^bright^Foxp3^−^ lymph node CD4 T cells and 5% of the same cells from conventional donors. The proportion of Ki-67^+^ MP lymph node cells was 38.7% in GF mice and 38% in conventional mice (Figure 2A). The absolute numbers of total lymph node CD4 T cells, of CD44^bright^ CD4 T cells and of Foxp3^−^ CD44^bright^ CD4 T cells were not different and thus there was no difference in the numbers of Ki-67^+^ or of BrdU^+^ cells. This finding was the case for both peripheral and mesenteric lymph node cells (Figure 2B and 2C). Thus, numbers and proliferative behavior of MP cells is similar in mice with very limited antigen-exposure (i.e., GF mice) to that in conventional mice, raising the possibility that a substantial proportion of MP cells in conventional mice may develop through a process other than foreign antigen-driven activation and expansion.

### Anti-MHC Class II Antibody Does Not Block Proliferation of MP Cells

**Figure 3:**
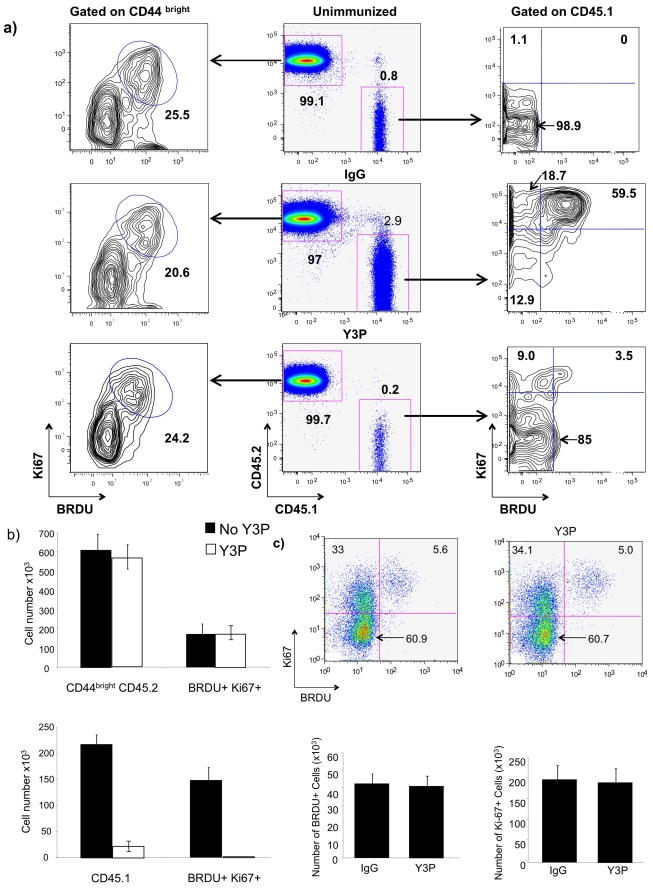
Effect of anti-MHC class II antibody (Y3P) on the proliferation of MP cells. (A) 2×10^6^ CD45.1 OT-II CD4 T cells were injected IP into CD45.2 FcγRγ^−/−^ B6 mice. 24 h later, mice were treated with Y3P (1.8 mg IP) or mouse immunoglobulin G (IgG), and 1 d later they were immunized IP with ovalbumin peptide (10 µg) plus LPS (25 µg) or LPS only. BrdU was given in drinking water from the time of immunization; 3 d later, lymph node cells were collected and stained with anti-CD45.1, anti-CD45.2, anti-CD44, anti-BrdU, and anti-Ki67. Contour plots were used because of the low number of cells available for analysis from mice treated with Y3P. (B) Numbers of CD45.1 and CD45.2 and of Ki-67^+^ BrdU^+^ cells from immunized control or Y3P-treated mice. (C) FcγRγ^−/−^ B6 mice were treated for 3 d with 1.8 mg of Y3P followed by a 6-h BrdU pulse (1 mg). Numbers in the quadrants represent the frequency of BrdU-positive and -negative and Ki-67-positive and -negative cells for an individual animal. Lower panels present means and SDs for numbers of BrdU^+^ and Ki-67^+^ cells among the three animals in each group.

One approach to evaluating the importance of antigen stimulation in T-cell dynamics is to determine whether proliferation can be blocked by anti-MHC class II antibodies [22]. To that end, we utilized FcγRγ^−/−^ mice so that the anti-class II antibody would not block T-cell responses by elimination of antigen-presenting cells. In such mice, anti-class II antibodies powerfully inhibit antigen-specific in vivo responses. We transferred CD45.1 OT-2 cells to CD45.2 FcγRγ^−/−^ C57BL/6 mice, treated the recipients with the anti-class II antibody Y3P (1.8 mg IP) and 1 d later immunized them with an ovalbumin peptide plus LPS. BrdU was given to these mice in drinking water from the time of immunization and mice were humanely killed 3 d later. Mice treated with mouse immunoglobulin G (IgG) rather than Y3P showed expansion of the transferred cells. 68.5% of these cells were BrdU^+^ and 78.2% were Ki-67^+^. By contrast, in the treated mice, there was no expansion of the transferred cells when compared to unimmunized mice and only 6% were BrdU^+^ and 12.5% Ki-67^+^ (Figure 3A and 3B). In the same animals, the frequency and number of MP cells that were BrdU^+^, Ki-67^+^ were not affected by treatment with Y3P (Figure 3A and 3B). In a separate experiment, in which BrdU was administered to nonimmunized FcγRγ^−/−^ mice for 6 h prior to humanely killing, Y3P had no effect on the frequency or numbers of BrdU^+^ or of Ki-67^+^ MP cells (Figure 3C).

### Anti–IL-7Rα Partially Inhibits MP Proliferation

**Figure 4:**
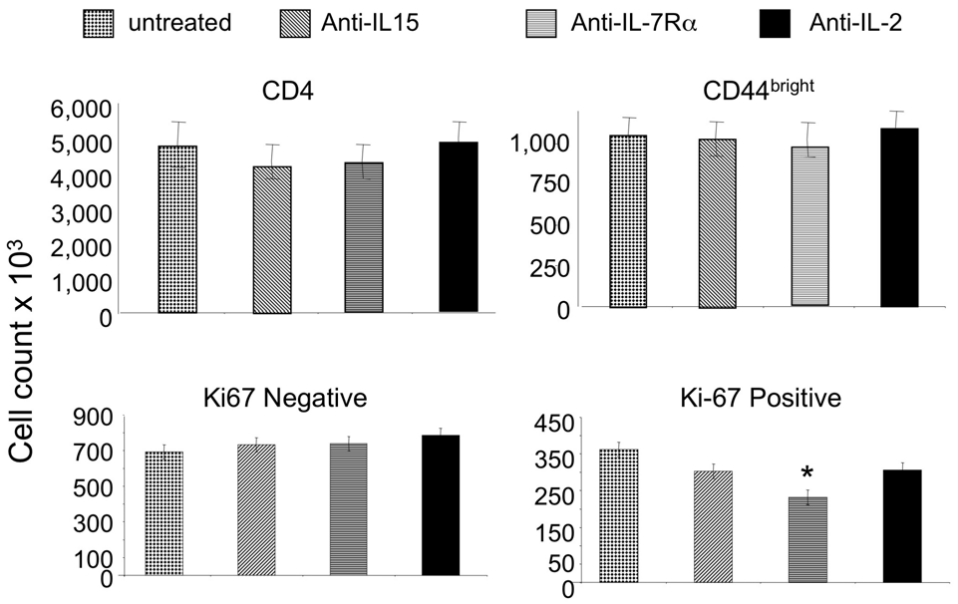
Effect of anti–IL-7Rα, anti–IL-15, and anti–IL-2 on in situ MP proliferation. Normal B6 mice were either untreated or received two doses of 0.5 mg of anti–IL-15, anti–IL-7Rα, or anti–IL-2 antibody spaced 3 d apart. Mice were humanely killed on day 7 and lymph node cell suspensions were stained with anti-CD4, anti-CD44, and anti–Ki-67. *p<0.05.

Normal C57BL/6 mice were either untreated or received anti–IL-15, anti–IL-7Rα, or anti–IL-2 antibody on day 1 and day 4 and were humanely killed on day 7. There was no effect on total numbers of CD44^bright^ cells in lymph nodes but the numbers of Ki-67^+^ cells was significantly reduced among recipients of anti–IL-7Rα (Figure 4), indicating that at least a portion of the proliferative response of MP cells depended on IL-7, or conceivably, thymic stromal lymphopoietin (TSLP). Neither anti–IL-15 nor anti–IL-2 had a significant effect.

### MP Cells Transferred to Rag2^−/−^ Recipients Undergo Anti-Class II Sensitive, Burst-like Proliferation

**Figure 5:**
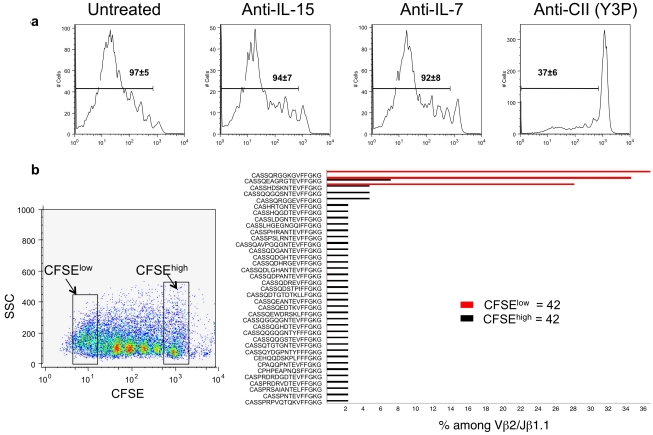
Effect of anti–IL-7, anti–IL-15, and anti-MHC class II (CII) on MP proliferation in Rag 2−/− mice. (A) 1 million CFSE-labeled MP CD4 T cells were transferred into Rag 2−/− mice that received a single dose of 0.5 mg of anti–IL-7, anti–IL-15, or 1.8 mg of Y3P or were untreated. On day 3, the mice received a second dose of anticytokine antibody or of Y3P. Mice were humanely killed on day 6 and single cell suspensions from lymph nodes were stained by anti-CD4 followed by flow cytometric analysis of CFSE dilution. Numbers inside the histograms represent the frequency of cells that have divided at least once (mean ± SD, from three mice per group). (B) 1 million CFSE-labeled MP CD4 T cells were transferred into Rag2−/− recipients. On day 3, animals were humanely killed and 50×10^3^ CFSE low and high cells were sorted, followed by PCR amplification using Vβ2 and Jβ1.1 primers (see Figure 6A). CDR3 sequences from the amplified DNA were plotted according to the frequency with which they appeared among the 42 sequences obtained from CFSE low and high Vβ2/Jβ1.1 cells, respectively.

Transfer of MP CD4 T cells into Rag2^−/−^ recipients results in burst-like proliferation such that the great majority of the cells present 6 to 7 d later have undergone 7 or more divisions, as judged by carboxyfluorescein diacetate succinimidyl ester (CFSE) dilution [5],[23]–[26]. We carried out such an experiment and confirmed that the majority of the transferred CD4 T cells present 6 d later had undergone multiple divisions. Neither anti–IL-15 nor anti–IL-7Rα had any inhibitory effect, but Y3P almost completely inhibited proliferation (Figure 5A), indicating that the expansion of cells in this lymphopenic setting required recognition of MHC and presumably of peptide/MHC complexes.

If MP cells are normally undergoing proliferation bursts, we reasoned that the dividing cells would have originated from relatively small cohorts of cells that would each go through many divisions. This should have the effect that the dividing cells would have relatively limited sequence diversity compared to nondividing MP cells in the same individual. To test whether this thesis is correct in a system in which we could be confident that cells had undergone multiple cell divisions, we transferred 1 million CFSE-labeled CD44^bright^ CD25^−^ CD4 T cells into Rag2^−/−^ C57BL/6 recipients. We humanely killed the mice 3 d after transfer so that the proportion of cells that had not divided would still be substantial; by 7 d, the cells that had divided multiple times completely dominate the distribution. We purified, by cell sorting, CD4^+^ CD44^bright^ cells that had completely diluted their CFSE and those that had retained their full amount of CFSE, implying they had not divided. We limited our sequence analysis to one Vβ/Jβ set, Vβ2/Jβ1.1. We chose Jβ1.1 since, as the most proximal Jβ, those TCRβ gene segments that have retained Jβ1.1 are very likely to be using it in their rearranged Vβ chain. Limiting the range of Vβs studied also allowed us to sample a larger fraction of the repertoire among those TCRs with a relatively small number of sequences than would have been the case had we tested all Vβs.

Among 42 sequences of transferred MP CD4 T cells that had not divided (CFSE^high^), 37 were unique; one sequence occurred three times, three occurred twice, and 33 but a single time (Figure 5B). By contrast, among 42 sequences from MP CD4 T cells that had divided seven or more times (CFSE^low^), there were only three unique sequences, occurring 12 (CASSHDSKNTEVFFGKG), 14 (CASSQEAGRGTEVFFGKG), and 16 (CASSQRGGKGVFFGKG) times, respectively. Interestingly, two of the sequences from the cells that divided multiple times were also found among the cells that had not divided, but in these cases they were represented only two or three times. This experiment validates our expectation that a cell population undergoing burst-like division should have a relatively limited repertoire that should be distinguishable from that of cells of a comparable phenotype that had not divided. It further indicates that only a subset of the MP cells undergo burst-like proliferation in a lymphopenic environment.

### MP Cell Division In Situ Is Largely Not Burst-like

**Figure 6:**
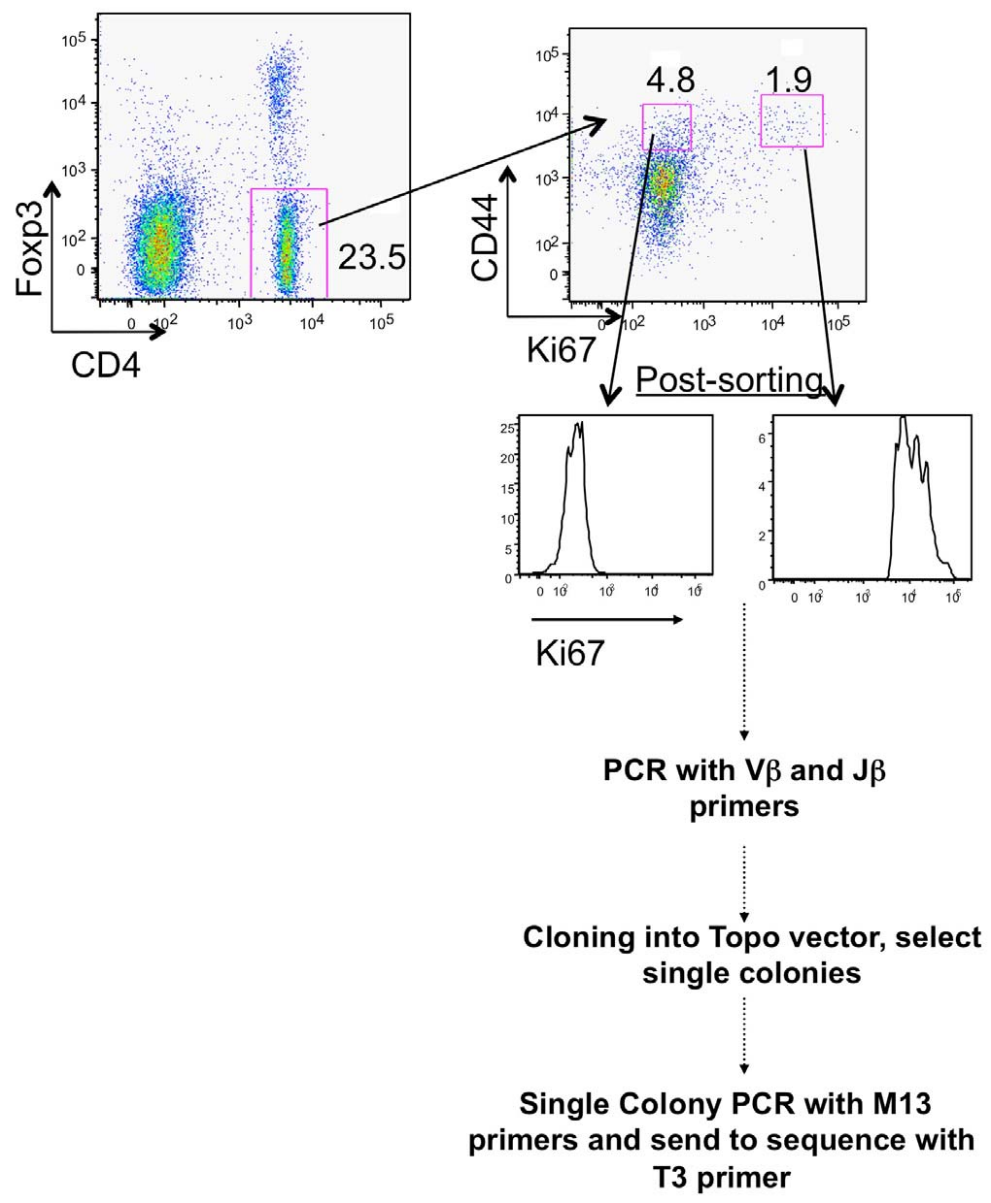
Sequence diversity among proliferating and nonproliferating Vβ2-Jβ1.1 and Vβ4-Jβ1.1 CD44^+^, Foxp3^−^ cells CD4 T cells (1). Experimental approach to analyze the CDR3 sequences of Vβ2-Jβ1.1 and Vβ4-Jβ1.1 CD4^+^ CD44^+^ Foxp3^−^, KI-67^bright^ and Ki-67^negative^ cells from individual mice. Cells were FACS sorted into lysate buffer containing proteinase K. One-third of the lysate was subjected to PCR with specific Vβ and Jβ1.1 primers (see Methods). PCR products were cloned into the Topo vector followed by bacterial transformation and single colony isolation. PCR with universal M13 primers were used on each colony to amplify the Vβ2-Jβ1.1 and Vβ4-Jβ1.1 gene segments followed by sequencing with the universal T3 primer.

**Figure 7:**
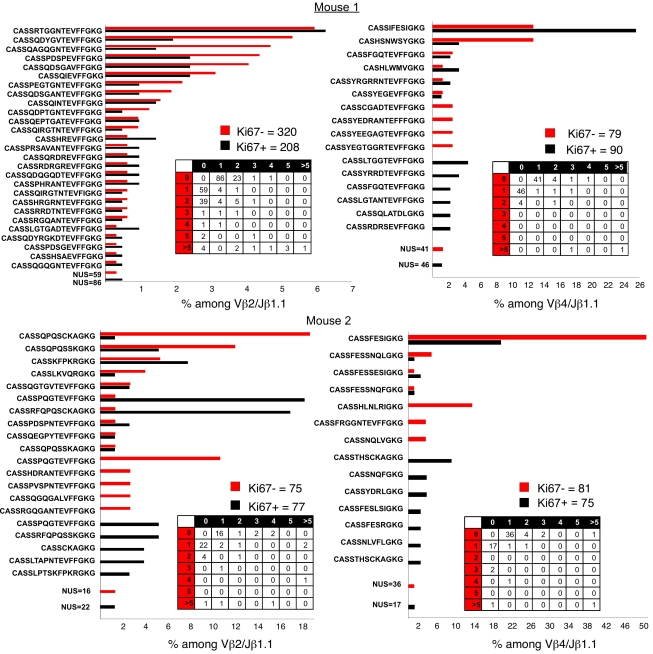
Sequence diversity among proliferating and nonproliferating Vβ2-Jβ1.1 and Vβ4-Jβ1.1 CD44^+^, Foxp3^−^ cells CD4 T cells (2). Sequence diversity within Vβ2-Jβ1.1 and Vβ4-Jβ1.1 Ki-67^bright^ and Ki-67^negative^ CD44^+^ CD4 T-cell populations. Shown is the percentage occurrence of a given sequence in two mice. *N* is indicated above each table for Ki-67^bright^ and Ki-67^negative^ cells. NUS indicates number of unique sequences that occur only once in either the Ki-67^bright^ and Ki-67^negative^ cells. Each table represents the numbers of sequences that occur once, twice, three times, four times, five times, and more than five times among the Ki-67^bright^ and Ki-67^negative^ populations.

To examine the repertoire complexity of proliferating MP cells in situ in comparison to that of nondividing MP cells, we took advantage of our observation that the MFI of Ki-67^+^ cells was highest among cells that had recently divided. We could not utilize pulse labeling with BrdU because the process of staining for BrdU expression requires the use of DNase, interfering with DNA sequence analysis. We sorted CD4^+^, Foxp3^−^, CD44^bright^, Ki-67^bright^ cells and CD4^+^, Foxp3^−^, CD44^bright^, Ki-67^negative^ cells and sequenced both Vβ2/Jβ1.1 and Vβ4/Jβ1.1 CDR3 segments from two individual mice (Figure 6). We obtained 320 Vβ2/Jβ1.1 sequences from the Ki-67^negative^ cells of mouse 1 and 208 sequences from the Ki-67^bright^ cells of this donor. We plotted sequences against their relative representation as indicated by their percentage frequency (Figure 7). We also listed the number of sequences that occurred one to five times or more than five times in the embedded table. Among the 320 sequences from the Ki-67^negative^ MP cells, 133 were unique. Of these, 103 occurred only once or twice. However, some sequences were much more frequent. The sequence CASSRTGGNTEVFFGKG comprised almost 6% of the Vβ2/Jβ1.1 sequences from Ki-67^ negative^ cells, and five other sequences constituted 3% or more of all the sequences. The pattern of sequence expression among the Ki-67^bright^ cells was quite similar to that of the Ki-67^negative^ cells (Figure 7). Of the 208 sequences, 138 were unique. Of these, 128 occurred once or twice. One sequence occurred ∼6% of the time. Interestingly, this was the same sequence (CASSRTGGNTEVFFGKG) that occurred most frequently among the Ki-67^negative^ cells, suggesting that it represents a clone whose dividing and nondividing members reflect prior clonal expansion, possibly from naïve cells, rather than a process of ongoing burst-like proliferation. However, we cannot exclude the possibility that the high frequency of this sequence represents a late phase of a clonal expansion episode when some of the cells have already stopped dividing and have lost Ki-67 expression.

We also sequenced Vβ4/Jβ1.1 CDR3 regions from the MP cells of the same mouse (Figure 7). In this case, we obtained 79 sequences from Ki-67^negative^ cells and 90 sequences from Ki-67^bright^ cells. The results were quite similar to those observed from the sequences of Vβ2/Jβ1.1 Ki-67^negative^ and Ki-67^bright^ cells. We had 56 unique sequences among those from Ki-67^negative^ cells; of these, 54 occurred once or twice. Two sequences were more common, both representing more than 12% of the total sequences. Among the 90 Vβ4/Jβ1.1 sequences from the Ki-67^bright^ cells, there were 53 unique sequences of which 48 occurred once or twice. One sequence (CASSIFESIGKG**)** constituted ∼25% of all the sequences; however, that sequence was one of the two that each constituted 12% of the sequences from the Ki-67^negative^ cells, implying that these dividing cells may be accounted for by the over-representation of the same particular clone and may not represent an ongoing burst-like expansion. As stated above, we cannot rule out the possibility that this represents a just-completed burst in which some cells have already ceased dividing. There are three sequences that occurred three times and one that occurred four times among the Ki-67^bright^ cells. Two of those had not occurred among the Ki-67^negative^ sequences and thus could conceivably represent burst-like proliferation. However, the majority of the dividing Ki-67^bright^ cells do not appear to have recently expanded from a precursor by multiple cell divisions.

We sequenced Vβ2/Jβ1.1 and Vβ4/Jβ1.1 CDR3 regions from Ki-67^negative^ and Ki-67^bright^ cells from a second donor (Figure 7, mouse 2). Although the results were generally similar, in this mouse, there were sequences among the Ki-67^bright^ cells that occurred multiple times but which were not found among the Ki-67^negative^ cells, suggesting they may represent burst-like proliferation. Indeed, among the Vβ2/Jβ1.1 sequences from mouse 2, two sequences comprised 18% and 17% of the sequences but were only observed once among the Ki-67^negative^ cells and two sequences observed in 5% of the Ki-67^bright^ sequences and not in the Ki-67^negative^. However, even among the 77 Vβ2/Jβ1.1 Ki-67^bright^ CDR3 sequences from this mouse, 31 were unique and 23 occurred only once or twice. Among the Vβ4/Jβ1.1 sequences, there was one from the Ki-67^bright^ cells that occurred in 9% of the sequences but was not found among the Ki-67^negative^ cells, suggesting that it may represent burst-like expansion. Here too, there were many unique sequences that occurred rarely. Of the 75 Vβ4/Jβ1.1 sequences from Ki-67^bright^ cells, there were 47 unique sequences of which 43 occurred only once or twice. Furthermore, it should be pointed out that we occasionally observe a dominant sequence among those from Ki-67^negative^ cells (in this mouse, 50% of the sequences from KI-67^negative^ are CASSFESIGKG) that is not (or is only infrequently) represented in the sequences from the Ki-67^bright^ cells.

Overall, we conclude that the complexity of Vβ2/Jβ1.1 and Vβ4/Jβ1.1 CDR3 sequences from Ki-67^bright^ cells cannot be distinguished from that of the Ki-67^negative^ cells. Estimating the maximum percentage of Ki-67^bright^ cells that could have been part of bursts from these data is nonetheless not simple. Taking the most inclusive view, it could be argued that all Ki-67^bright^ sequences that are represented many times should be considered as having originated from burst-like clonal expansion during the period immediately before the mice were humanely killed. To obtain an estimate of the frequency of such events, we summed all the sequences that occurred four or more times in the Ki-67^bright^ cells. In the four groups studied, there were 119 sequences among those that occurred four or more times. Since the total number of Ki-67^bright^ sequences analyzed was 450, this implies that ∼25% of the sequences may represent Ki-67^bright^ cells that were part of burst-like clonal expansion. If we exclude those 88 sequences that occurred multiple times in both the Ki-67^negative^ and Ki-67^bright^ groups, then the proportion of dividing cells that are part of burst-like proliferation is ∼7%. Thus, the majority of dividing cells do not appear to be part of an ongoing process of burst-like clonal expansion from a limited number of precursors, which was the case when we examined the burst-like division of the Vβ2/Jβ1.1 MP cell population that occurred upon transfer to severely lymphopenic recipients.

### TCR Vβ Sequences from GF MP Cells Resemble Those from Conventional Donors

**Figure 8:**
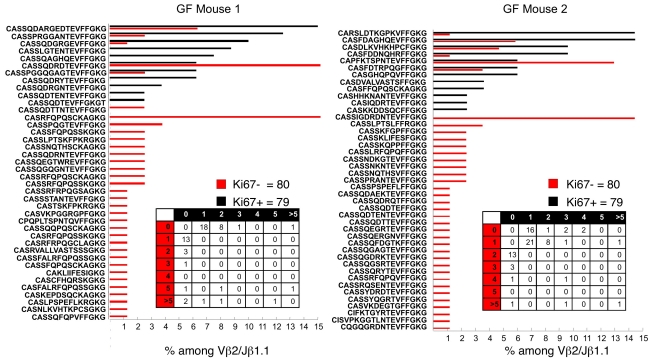
Sequence diversity among proliferating and nonproliferating Vβ2-Jβ1.1 cells in B6 GF mice. Sequence diversity among Ki-67^bright^ and Ki-67^negative^ Vβ2-Jβ1 CD44^+^ CD4 T-cell populations. Shown is the percentage of times an individual sequence occurred in two GF mice.

We also examined sequences from Ki-67^negative^ and Ki-67^bright^ Vβ2/Jβ1.1 CD44^bright^ cells from lymph nodes of GF mice. Overall, the patterns of sequence distribution were remarkably similar to those of conventional mice (Figure 8). There were large numbers of unique sequences, most of which were represented only once or twice. There were some CDR3 sequences that did occur relatively frequently among the Ki-67^bright^ cells and were also frequent among the Ki-67^negative^ cells. In each mouse, one sequence was represented frequently among the Ki-67^bright^ cells but was not observed among the Ki-67^negative^ cells. In mouse one, it constituted ∼26% of the Vβ2/Jβ1.1CDR3 sequences from the Ki-67^bright^ cells; in mouse 2, it constituted 20% of the sequences. Thus, a considerable minority of the proliferation of the GF MP cells may have derived by burst-like expansion.

While the sample size of sequences from the GF donors was relatively small, they showed substantial diversity in both the Ki-67^negative^ and Ki-67^bright^ cells, suggesting that in the GF mice, the CD44^bright^ cells have not arisen by differentiation and expansion of cells with a very limited TCR repertoire.

## Discussion

MP CD4 T cells from normal mice (i.e., mice housed in specific pathogen-free facilities) proliferate quite rapidly. BrdU labeling reveals that ∼10% of these cells from lymph nodes take up BrdU in a single day and more than 30% of MP cells are Ki-67^+^; on the basis of our estimate that the great majority of Ki-67^+^ cells have divided within the past 3 d, this implies that more than 30% of lymph node MP cells divide in 3 d, a result that is confirmed by more extended BrdU labeling. The frequency of Ki-67^bright^ MP cells is somewhat less in the spleen. By contrast, NP cells take up BrdU at ∼0.1% per day and very few are Ki-67^+^.

What drives the rapid proliferation of the MP cells has been a matter of uncertainty. Some have concluded that their proliferation is driven by TCR engagement on the basis of transfer to lymphopenic hosts, where it is observed that by 7 d after transfer the majority of the surviving cells have divided seven times or more. Zamoyska and colleagues [25] and Leignadier et al. [27] have used a tetracycline/off system to delete TCR from mature T cells. In both instances, deleting TCR resulted in a substantial diminution in the proportion of CD44^bright^ CD4 T cells that had gone through multiple divisions when transferred to lymphopenic recipients. Similarly, anti-MHC class II antibody blocked expansion of CD4 T cells introduced into neonatal recipients [23], and we showed here that the rapid proliferation of MP CD4 T cells introduced into Rag2^−/−^ recipients was completely inhibited by the anti-MHC class II antibody Y3P. Furthermore, Surh and colleagues reported that the rapid proliferation of CD4 T cells that occurred when these cells were introduced into *scid* mice was largely lost if the *scid* recipients were GF [28]. As a group, these observations clearly indicate that in severely lymphopenic settings, expansion of MP cells depends on TCR recognition of peptide/MHC complexes.

The results we present here indicate that only a portion of the transferred MP cells undergo this striking proliferation. When we sequenced CDR3 segments from the Vβ2/Jβ1.1 TCRs 3 d after transfer of MP cells into Rag2^−/−^ recipients, we found only three sequences among the rapidly dividing cells, whereas there were 37 sequences among those that had not divided, implying that the rapid proliferation was a property of a limited set of cells among the transferred MP population. This result is consistent with the observation that naïve CD4 T cells from most TCR transgenic donors fail to rapidly proliferate on transfer to lymphopenic recipients [1],[24],[29] and on our immunoscope analysis of TCR Vβ complexity in Rag2^−/−^ recipients of numbers of CD4 T cells varying from 10 million to 10,000 [29] suggesting that only ∼3% of the transferred cells undergo rapid expansion. It is interesting that two of the sequences represented frequently in the dividing cells were also found in the nondividing population, implying that not all cells of the same specificity are stimulated in a lymphopenic environment.

However, the results obtained by the study of transfer to lymphopenic environments do not appear to be a valid representation of the mechanisms underlying the rapid proliferation of MP cells in situ. Indeed, survival of MP cells in lymphocyte-sufficient settings has been reported to not require expression of TCRs. Furthermore, there is a large literature demonstrating that survival of antigen-specific memory cells arising during immunization does not require TCR engagement, but rather depends upon the availability of cytokines, particularly IL-7 and IL-15 [10]–[12],[23],[24]. However, we wish to point out that the analysis of antigen-specific memory cells emerging from intentional immunization may not necessarily represent what governs the proliferative behavior of MP cells.

Indeed, both the work presented here and recent studies analyzing antigen-specific CD4 memory T cells at varying times after priming show that antigen-specific memory cells emerging from intentional immunization divide relatively slowly compared to MP cells. Lenz at al. [7] infected mice with LCMV. 50 d later, a 7-d exposure to BrdU resulted in only 15% of BrdU^+^ spleen cells among those capable of producing interferon gamma (IFNγ) in response to challenge with two different LCMV peptides. Purton et al. [5] transferred TCR transgenic SMARTA CD4 T cells, specific for an LCMV epitope, into C57BL/6 mice that were then infected with LCMV. 72 d after infection, 12% of the transgenic cells took up BrdU during a 5-d labeling period. Jenkins and colleagues [6] infected mice with *Listeria monocyogenes* expressing an ovalbumin peptide (LM2W1S). 40 d after infection, the mice received BrdU for 14 d; among spleen and lymph node CD4 T cells capable of binding an ovalbumin tetramer, only 11.5% were BrdU^+^.

Our results are consistent with these reports. We infected C57BL/6 mice with LCMV. Fifteen and 60 d later, the frequency of Ki-67^+^ cells among tetramer^+^ cells was measured. At 15 d, 8% of the CD44^bright^ tetramer^+^ cells were Ki-67^+^; at 60 d, ∼7%. Collectively, these studies indicate that after the expansion phase following immunization is complete, antigen-specific CD44^bright^ CD4 T cells divide at a rate of <1% to ∼2.5% per day. The possibility that MP cells and authentic memory cells might represent distinct cell types, or rather that the MP pool contains both authentic memory cells and another cell population, was also suggested by our prior study in SW GF mice that showed that their MP CD4 T cells proliferated at a rate similar to MP cells from conventional donors [3]. We have examined this point in greater detail here in GF and conventional C57BL/6 mice and confirm that the proportion and absolute number of non-Treg CD44^bright^ CD4 T cells from peripheral and mesenteric lymph nodes of GF mice are similar to those from conventional mice as are the proportion and number of proliferating MP cells. It should also be pointed out that prior studies of GF mice maintained on elemental diets (i.e., antigen-free mice) had shown the presence of substantial numbers of activated CD4 T cells, equivalent in frequency to those in conventional mice [30]–[32]. While these studies were carried out before the availability of the reagents now used to classify MP cells, they strongly suggest that antigen-free mice have similar numbers of MP CD4 T cells as do conventional mice and thus support the concept that foreign (including commensal) antigens are not critical to the emergence of the majority of MP cells.

Here we have shown that the in situ proliferation of MP cells is not inhibited by anti-MHC class II antibody, using a reagent that strikingly inhibits the proliferation of antigen-specific cells in response to antigen challenge and that blocks the rapid proliferation of MP cells transferred to lymphopenic recipients. Rather, we observe that anti–IL-7Rα antibody diminishes, but does not abolish, proliferation of MP cells, implying that IL-7 or TSLP plays a role in this proliferation.

An alternative way to examine the issue of whether the rapid proliferation of these cells represents an antigen-driven response, during which one would anticipate that limited numbers of precursors give rise to bursts consisting of multiple divisions, is to examine the TCR sequence diversity of proliferating MP cells and to compare that to the sequence diversity of quiescent MP cells. If MP proliferation was primarily due to burst-like clonal expansion stimulated by exposure to antigen, it would be expected that the sequence diversity of the proliferating cells would be substantially less than that of the quiescent cells. Indeed, when we studied proliferating and nonproliferating MP cells in lymphopenic recipients, this is precisely what we observed.

We examined CDR3 sequences from Vβ2/Jβ1.1 and Vβ4/Jβ1.1 MP CD4 T cells that were Ki-67^bright^ or Ki-67^negative^. We chose to limit our study to these two TCR Vβ sets so that we could sample a larger proportion of these defined subrepertoires than we could with the same number of sequences of total CD44^bright^ Ki-67^bright^ cells. As an estimate of the number of cells under study, we used the following considerations. The total number of lymph node CD4 T cells is ∼8 million. ∼15% of these cells are CD44^bright^, or ∼1.2 million. Of these, ∼half are CD25^+^, so that MP cells constitute ∼600,000; ∼5% express Vβ2 or Vβ 4, or ∼30,000. Of the Vβ2 or Vβ 4 expressing cells, ∼10% are Jβ1.1, or ∼3,000. The Ki67^bright^ cells are ∼10% of the MP cells so that the total number of Ki-67^bright^ Vβ2/Jβ1.1 or Vβ4/Jβ1.1 MP cells in all the lymph nodes of the animal is ∼300. Thus, the maximum number of unique sequences would be 300 in this cell population; this would be substantially less if repetitive sequences existing among these cells, which would be anticipated on the basis of the likelihood that the generation of MP cells from naïve precursors involved clonal expansion. Thus, in our initial analysis, involving >200 sequences from the CD44^bright^ Ki-67^bright^ Vβ2/Jβ1.1 cells of mouse 1, our sample, while not complete, is quite substantial. Even the samples of 70 to 80 sequences in the other cases are sufficient to provide useful information about complexity, as judged by our observations of multiply repeated sequences.

A further point is our reliance on CDR3 sequences from the β chain of the TCR as a clonal marker. It is possible that we have overestimated the frequency of repeats since there may be occasions in which the same Vβ is used with different Vα's, but we suspect that in the vast majority of cases the CDR3 sequence of the TCR β chain is indeed a clonal marker.

Our results indicate that the distribution of sequences in the Ki-67^bright^ and Ki-67^negative^ populations was not markedly different. If we made the assumption that any sequences that occurred many times among the Ki-67^bright^ cells represented cells that had recently been in a burst-like expansion, then ∼25% of the Ki-67^bright^ cells would be judged to be in such bursts. To obtain this estimate we arbitrarily assigned any sequence that occurred four or more times among the Ki-67^bright^ cells to the set that occurred “many” times. However, this could easily be an overestimate depending on how one interprets those instances in which a similarly high frequency of the same sequence was found among Ki-67^negative^ cells. On the one hand, this might reflect a large clone in which cell division occurred on a stochastic basis so that the frequency of the clone was similar among the dividing and nondividing cells. If we make this assumption, then the proportion of Ki-67^bright^ cells that were in bursts becomes ∼7%. Alternatively, instances in which a sequence found in the Ki-67^bright^ cells was equally (or over-represented) among the Ki-67^negative^ cells may represent the late-phase of a burst episode in which a portion of the cells had already stopped dividing and lost Ki-67 expression but others continued to divide. Overall, we conclude that the sequence data are consistent with a minority of the Ki-67^bright^ cells being part of burst; whether that minority is small or considerable cannot easily be determined. However, when taken together with the failure of anti-class II antibody to block proliferation of MP cells, it is reasonable to conclude that the proportion of Ki-67^bright^ cells that are part of burst-like expansion is quite small.

There may be circumstances in which clonal expansion/burst-like antigen-driven proliferation plays a much great role than is found among MP cells from normal mice. It has been argued that the dynamics of MP cells from SIV-infected macaques, in which proliferative rates are far higher than proliferative rates of MP cells from noninfected macaques, is best explained by multiple overlapping burst episodes of several cell divisions occurring within a brief period of time [17],[18]. These cells, which exist in a highly inflammatory setting, may well show enhanced sensitivity to their cognate antigens or to self-peptide/MHC complexes. A detailed analysis of their sequence complexity and of the complexity of MP cells derived from chronically infected individuals or individuals that were in a state of chronic inflammation would help to clarify this point.

As discussed above, we found that GF and conventional C57BL/6 mice have comparable numbers of MP CD4 T cells in their peripheral and mesenteric lymph nodes and these cells display similar proliferative rates. We sequenced CDR3 gene segments from Vβ2/Jβ1.1 Ki67^bright^ and Ki-67^negative^ MP cells of two GF mice and found that they, like the comparable cells from conventional mice, were very similar in their sequence diversity. One could argue that GF mice are not antigen free, although there are reports that antigen-free mice show normal numbers of “activated” CD4 T cells. Nonetheless, there can be little doubt that GF mice have a much reduced antigenic experience. If the MP cells of GF mice represent clonal expansions because of an extremely limited set of naïve cells responding to a comparably limited set of antigenic stimuli, it would be anticipated that the MP cells from GF mice would have a much more restricted repertoire than MP cells from conventional mice. While our limited number of sequences may not be sufficient to reach a definitive conclusion on this point, there does not appear to be any major difference in the degree of diversity of the Ki-67^bright^ or Ki-67^negative^ MP cells from the two sets of donors.

On the basis of these observations, we propose that MP cells may be a more diverse population than had been considered and that only some of these cells may have emerged by exposure to conventional foreign antigens. The maintenance of most MP cells and their rapid proliferative rate appear to be largely dependent on cytokines. The origin of the infrequently occurring proliferation bursts remains to be clarified, but an obvious possibility is through recognition and response to self-peptides on competent antigen-presenting cells. It should be pointed out that diversity in CD8 T cells has also been described, with one population being designated “bystanders” and that such cells take on a memory phenotype in mice deficient in the transcription factor KLF2, the signaling kinase itk, or the histone acetyltransferase CBP [33]. Whether such “bystander” CD8 cells bear a relationship to the rapidly dividing CD4 MP cells discussed here remains to be determined.

Overall, one may ask what is the function of the large set of MP cells in normal mice? We have proposed [23],[34]–[37] that they represent a pool of cells capable of making a rapid effector response to cross-reactive antigens of pathogens during a period in which the naïve cells proliferate and differentiate. MP cells might play an even more important role in instances in which naïve cells are limiting and no “authentic” memory cells are specific for an introduced pathogen, such as might be the case in aged individuals. Devising models in which these cells are absent will be essential to testing their function. Finally, why proliferative rates of authentic memory and MP cells are different is not clear.

## Materials and Methods

### Mice and Infection

C57BL/6 (B6), B6 Rag2^−/−^, B6 FcγRγchain^−/−^, and OT-II CD45.1 mice were obtained from the National Institute of Allergy and Infectious Diseases (NIAID) contract facility at Taconic Farms. GF mice were maintained at the NIAID GF facility. All other mice were maintained under pathogen-free conditions in NIAID animal facilities. Mice infected with LCMV were inoculated IP with 2×10^5^ PFU Armstrong strain. The care and handling of the animals used in our studies was in accordance with the guidelines of the National Institutes of Health (NIH) Animal Care and Use Committee.

### Flow Cytometry and Antibodies

Y3P was obtained from Harlan Bioproducts. Antibodies to IL-15 (5H4), IL-2 (S4B6), CD127 (IL-7Rα; SB/14), CD4 (pacific blue; RM4-5), Ki-67 (PE; B56), Vβ2 (FITC; B20.6), Vβ4 (FITC; KT4) were purchased from BD Biosciences. Anti-CD44 (Alexa-700; IM7) and anti-Foxp3 (PE; NRRF-30) were purchased from eBioscience. The detection of BrdU was carried out according to instructions in the kit provided by BD Biosciences. The I-A^b^-GP-66-77 tetramer that recognizes receptors for an immunodominant LCMV epitope was provided by the NIH tetramer facility (Emory Vaccine Center). All flow cytometry analyses were performed using an LSR-II (BD Biosciences).

### Adoptive Transfer

Inguinal, axillary, cervical, and mesenteric CD4 MP lymph node T cells were obtained by sorting on a FACSAria (BD Biosciences). Purity was >99% CD4, CD44^+^ or, depending on the experiment, CD4, CD44^+^, Foxp3^−^. In some experiments, sorted cells were labeled with CFSE (Molecular Probes) at a final concentration of 1.25 µmol and transferred IP into recipient mice.

### CDR3 Sequencing

From 10^5^ to 0.5×10^5^ KI-67 negative and bright CD4 CD44^bright^, Foxp3^−^ T cells were FACS-sorted into FCS. Cells were resuspended in lysis buffer (20 mmol Tris-HCl, pH 7.5, 150 mmol NaCl) with 4 µg/ml proteinase K (Fermentas), incubated at 56°C for 50 min and then at 95°C for 10 min. Volumes were adjusted to 30 µl; 10 µl were used to amplify the Vβ2/Jβ1.1 and Vβ4/Jβ1.1 CDR3s with the following primers: Vβ2 5′ CAGTCGCTTCCAACCTCAAAGTTC′ or Vβ4 5′ CGATAAAGCTCATTTGAATCTTCGAATC and 3′ Jβ1.1 AGCTTTACAACTGTGAGTCTGGTTCCTTTACC using 35 PCR cycles of 45 s at 95°C, 45 s at 57°C, and 45 s at 72°C. The PCR products were cloned into the TOPO blunt end vector (Invitrogen) and bacteria were transformed. Single colonies were isolated and suspended into 10 µl of water. PCR was carried out on 3 µl of bacterial suspension from single colonies using the universal M13 primers. PCR products were sequenced by Agencourt (Beckman Coulter) using universal T3 primer. The rate of readable sequences was 70% to 80%.

### Estimation of Frequency of Jβ1.1 Usage

Lymph node cells were stained with anti-CD4, anti-Vβ2, or anti-Vβ4 followed by single-cell sorting into 96-well plates. Plates were heated to 95°C for 3 min followed by a first round PCR of 40 cycles (45 s 95°C, 45 s at 57°C, 45 s at 72°C) using the 5′ Vβ2 or Vβ4 primers and a 3′ Jβ2.7 AGGCTCACGGTTTTAG primer. 3 µl of the first PCR product were subjected to a second PCR (25 cycle of 45 s at 95°C, 45 s at 57°C, 45 s at 72°C) using the Vβ2 or Vβ4 primers and the 3′ Jβ1.1 primer. PCR products were obtained in eight and seven wells out of 96 for Vβ2 or Vβ4, respectively, indicating a frequency for each of ∼10%.

### Analysis of Vβ2/Jβ1.1 and Vβ4/Jβ1.1 Sequences

Analysis of the CDR3 sequences was performed using Matlab as a platform to generate a program to analyze the sequences.

## Abbreviations

CFSE: carboxyfluorescein diacetate succinimidyl ester
GF: germ-free
IP: intraperitoneal
LCMV: lymphocytic choriomeningitis virus
MP: memory phenotype
NP: naïve-phenotype
SD: standard deviation
TCR: T-cell receptor

## References

[1] Surh, CD, Sprent J (2008). Homeostasis of naïve and memory T cells.. Immunity.

[2] Tough, DF, Sprent J (1994). Turnover of naïve- and memory-phenotype T cells.. J Exp Med.

[3] Min B, Thornton A, Caucheteux S. M, Younes S. A, Oh K (2007). Gut flora antigens are not important in the maintenance of regulatory T cellheterogeneity and homeostasis.. Eur J Immunol.

[4] Li J, Huston G, Swain S. L (2003). IL-7 promotes the transition of CD4 effectors to persistent memory cells.. J Exp Med.

[5] Purton J. F, Tan J. T, Rubinstein M. P, Kim D. M, Sprent J (2007). Antiviral CD4+ memory T cells are IL-15 dependent.. J Exp Med.

[6] Pepper M, Linehan J. L, Pagan A. J, Zell T, Dileepan T (2010). Different routes of bacterial infection induce long-lived TH1 memory cells and short-lived TH17 cells.. Nat Immunol.

[7] Lenz D. C, Kurz S. K, Lemmens E, Schoenberger S. P, Sprent J (2004). IL-7 regulates basal homeostatic proliferation of antiviral CD4+ T cell memory.. Proc Natl Acad Sci U S A.

[8] Swain S. L, Hu H, Huston G (1999). Class II-independent generation of CD4 memory T cells from effectors.. Science.

[9] Boyman O, Purton J. F, Surh C. D, Sprent J (2007). Cytokines and T-cell homeostasis.. Curr Opin Immunol.

[10] Geginat J, Campagnaro S, Sallusto F, Lanzavecchia A (2002). TCR-independent proliferation and differentiation of human CD4+ T cell subsets induced by cytokines.. Adv Exp Med Biol.

[11] Lohning M, Hegazy A. N, Pinschewer D. D, Busse D, Lang K. S (2008). Long-lived virus-reactive memory T cells Generated from purified cytokine-secreting T helper type 1 and type 2 effectors.. J Exp Med.

[12] Singh V, Gowthaman U, Jain S, Parihar P, Banskar S (2010). Coadministration of interleukins 7 and 15 with bacilli Calmette-Guerin mounts enduring T cell memory response against Mycobacterium tuberculosis.. J Infect Dis.

[13] Tan J. T, Ernst B, Kieper W. C, LeRoy E, Sprent J (2002). Interleukin (IL)-15 and IL-7 jointly regulate homeostatic proliferation of memory phenotype CD8+ cells but are not required for memory phenotype CD4+ cells.. J Exp Med.

[14] Tokoyoda K, Zehentmeier S, Hegazy A. N, Albrecht I, Grun J. R (2009). Professional memory CD4+ T lymphocytes preferentially reside and rest in the bone marrow.. Immunity.

[15] Combadere B, Blanc C, Li T, Carcelain G, Delaugerre C (2000). CD4+Ki67+ lymphocytes in HIV-infected patients are Effector T cells accumulated in the G1 phase of the cell cycle.. Eur J Immunol.

[16] Picker L. J, Hagen S. J, Lum R, Reed-Inderbitzin E. F, Daly L. M (2004). Insufficient production and tissue delivery of CD4+ memory T cells in rapidly progressive simian immunodeficiency virus infection.. J Exp Med.

[17] Grossman Z, Meier-Schellersheim M, Paul W. E, Picker L. J (2006). Pathogenesis of HIV infection: what the virus spares is as important as what it destroys.. Nat Med.

[18] Grossman Z, Picker L. J (2008). Pathogenic mechanisms in simian immunodeficiency virus infection.. Curr Opin HIV AIDS.

[19] Douek D. C, Roederer M, Koup R. A (2009). Emerging concepts in the immunopathogenesis of AIDS.. Annu Rev Med.

[20] Okoye A, Meier-Schellersheim M, Brenchley J. M, Hagen S. I, Walker J. M (2007). Progressive CD4+ central memory T cell decline results in CD4+ effector memory insufficiency and overt disease in chronic SIV infection.. J Exp Med.

[21] Pitcher C. J, Hagen S. I, Walker J. M, Lum R, Mitchell B. L (2002). Development and homeostasis of T cell memory in rhesus macaque.. J Immunol.

[22] Dorfman J. R, Stefanova I, Yasutomo K, Germain R. N (2000). CD4+ T cell survival is not directly linked to self-MHC-induced TCR signaling.. Nat Immunol.

[23] Min B, McHugh R, Sempowski G. D, Mackall C, Foucras G (2003). Neonates support lymphopenia-induced proliferation.. Immunity.

[24] Min B, Paul W. E (2005). Endogenous proliferation: burst-like CD4 T cell proliferation in lymphopenic settings.. Semin Immunol.

[25] Seddon B, Tomlinson P, Zamoyska R (2003). Interleukin 7 and T cell receptor signals regulate homeostasis of CD4 memory cells.. Nat Immunol.

[26] Surh C. D, Boyman O, Purton J. F, Sprent J (2006). Homeostasis of memory T cells.. Immunol Rev.

[27] Leignadier J, Hardy M. P, Cloutier M, Rooney J, Labrecque N (2008). Memory T-lymphocyte survival does not require T-cell receptor expression.. Proc Natl Acad Sci U S A.

[28] Kieper W. C, Troy A, Burghardt J. T, Ramsey C, Lee J. Y (2005). Recent immune status determines the source of antigens that drive homeostatic T cell expansion.. J Immunol.

[29] Min B, Foucras G, Meier-Schellersheim M, Paul W. E (2004). Spontaneous proliferation, a response of naïve CD4 T cells determined by the diversity of the memory cell repertoire.. Proc Natl Acad Sci U S A.

[30] Bruno L, von Boehmer H, Kirberg J (1996). Cell divisions in the compartment of naïve and memory T lymphocytes.. Eur J Immunol.

[31] Cederbom, L, Bandeira A, Coutinho A, Ivars F (1998). Naturally activated CD4+ T cells are highly enriched for cytokine-producing cells.. Eur J Immunol.

[32] Jiang H. Q, Thurnheer M. C, Zuercher A. W, Boiko N. V, Bos N. A (2004). Interactions of commensal gut microbes with subsets of B- and T-cells in the murine host.. Vaccine.

[33] Weinreich M. A, Odumade O. A, Jameson S. C, Hogquist K. A (2010). T cells expressing the transcription factor PLZF regulate the development of memory-like CD8+ T cells.. Nat Immunol.

[34] Haluszczak C, Akue A. D, Hamilton S. E, Johnson L. D, Pujanauski L (2009). The antigen-specific CD8+ T cell repertoire in unimmunized mice includes memory phenotype cells bearing markers of homeostatic expansion.. J Exp Med.

[35] Grossman Z, Min B, Meier-Schellersheim M, Paul W. E (2004). Concomitant regulation of T-cell activation and homeostasis.. Nat Rev Immunol.

[36] Grossman Z, Paul W. E (2000). Self-tolerance: context dependent tuning of T cell antigen recognition.. Semin Immunol.

[37] Grossman Z, Paul W. E (2001). Autoreactivity, dynamic tuning and selectivity.. Curr Opin Immunol.

